# Low Vaccine Coverage and Factors Associated with Incomplete Childhood Immunization in Racial/Ethnic Minorities and Rural Groups, Central Brazil

**DOI:** 10.3390/vaccines11040838

**Published:** 2023-04-13

**Authors:** Juliana de Oliveira Roque e Lima, Valéria Pagotto, Bárbara Souza Rocha, Paulo Sérgio Scalize, Rafael Alves Guimarães, Márcio Dias de Lima, Leandro Nascimento da Silva, Michele Dias da Silva Oliveira, Winny Éveny Alves Moura, Sheila Araújo Teles, Claci Fátima Weirich Rosso, Karlla Antonieta Amorim Caetano

**Affiliations:** 1 Faculty of Nursing, Federal University of Goiás, Goiânia 74605-080, GO, Brazil; 2 School of Civil and Environmental Engineering, Federal University of Goiás, Goiânia 74605-220, GO, Brazil; 3 Institute of Tropical Pathology and Public Health, Federal University of Goiás, Goiânia 74605-050, GO, Brazil; 4 Federal Institute of Education, Science, and Technology of Goiás, Goiânia 74055-110, GO, Brazil; 5 Municipal Health Department, Goiânia 74884-092, GO, Brazil

**Keywords:** vaccination coverage, rural population, immunization schedules

## Abstract

Discrimination and limited access to healthcare services in remote areas can affect vaccination coverage. Therefore, this study aimed to estimate vaccination coverage for children living in quilombola communities and rural settlements in the central region of Brazil during their first year of life and to analyze the factors associated with incomplete vaccination. An analytical cross-sectional study was conducted on children born between 2015 and 2017. The percentage of children who received all vaccines recommended by the National Immunization Program in Brazil by 11 months and 29 days was used to calculate immunization coverage. Children who received the following vaccines were considered as having a complete basic vaccination schedule: one dose of BCG; three doses of Hepatitis B, of Diphtheria-Tetanus-Pertussis (DPT), of *Haemophilus influenzae* type b (Hib), and of Poliovirus (Polio); two doses of Rotavirus, of 10-valent pneumococcal (PCV10), and of Serogroup C meningococcal conjugate (MenC); and one dose of Yellow Fever (YF). Measles-mumps-rubella (MMR) and other doses recommended at or after 12 months were not included. Consolidated logistic regression was used to identify factors associated with incomplete vaccination coverage. Overall vaccination coverage was 52.8% (95% CI: 45.5–59.9%) and ranged from 70.4% for the Yellow Fever vaccine to 78.3% for the Rotavirus vaccine, with no significant differences between the quilombola and settler groups. Notably, the likelihood of incomplete general vaccination coverage was higher among children who did not receive a visit from a healthcare professional. Urgent strategies are required to achieve and ensure health equity for this unique and traditionally distinct group with low vaccination coverage.

## 1. Introduction

The World Health Organization (WHO) has addressed immunization inequity, and global efforts to promote vaccine access are encouraged [1]. In 2021, 25 million children worldwide (19%) did not receive basic vaccines, such one or more doses of the Diphtheria-Tetanus-Pertussis vaccine (DTP). This number of undervaccinated children has increased by 5.9 million since 2019. Countries such as Angola, Brazil, the Democratic Republic of Congo, Ethiopia, India, Indonesia, Myanmar, Nigeria, Pakistan, and the Philippines comprise 60% of these children [2].

In Brazil, the National Immunization Program (NIP) was established in 1973, and it is considered an international benchmark due to its scope and performance, offering most WHO-recommended vaccines free of charge [3,4]. However, the country still faces challenges in achieving the expected worldwide vaccine coverage [5].

Incomplete childhood vaccination may be associated with demographic, socioeconomic, and policy-related factors [6]. Additionally, gender inequalities, ethnic discrimination, and limited access to health services in remote areas may also affect vaccination coverage [7].

In rural Brazil, significant inequalities are observed regarding urban environments and diverse races, peoples, and cultures. Settlers are rural groups that rely on family agricultural production, demanding agrarian reform [8]. Quilombolas are ethnic groups distributed throughout Brazil, residing in rural or urban areas, predominantly black, with their own historical ties [9]. Settlers and traditional quilombola communities stand out in this scenario, characterized by cultural isolation, popular struggles of resistance, and deprioritization. Little is known about their living and health conditions [10].

To date, there are no data on vaccination coverage for children residing in settlements and quilombola areas in Brazil. Therefore, situational diagnoses regarding access and factors associated with vaccination in vulnerable areas of the country are critical. These data can guide the development of more effective actions, informing decision-making in public policies and promoting universal access to health services.

This study aims to estimate vaccination coverage for the complete basic schedule during the first year (Table 1) and analyze the factors associated with incomplete vaccination in settled and quilombola children in the state of Goiás, Brazil, in response to the gaps in vaccination for children in rural Brazil.

## 2. Materials and Methods

### 2.1. Study Design

A cross-sectional retrospective cohort analytical study was conducted in 36 municipalities in the state of Goiás. This investigation is part of the “Sanitation and Environmental Health in Rural and Traditional Communities of Goiás-SanRural Project” matrix project. The SanRural Project aims to promote knowledge about sanitation conditions and the environmental health of settled and traditional communities, such as riverside communities and remnants of quilombos.

### 2.2. Context

The State of Goiás is located in the Center-West Region of Brazil and comprises 246 municipalities distributed in five mesoregions (Centro Goiano, East Goiano, Northwest Goiano, North Goiano, and South Goiano). Goiás is the most populous state in the Midwest region and has the ninth-largest economy in the country. Agriculture is the main economic activity in the state and one of the main factors responsible for the rapid process of agro-industrialization in Goiás. The state of Goiás has an estimated population of 6 million people, with approximately 10% residing in rural areas [11]. According to the IBGE, 117 quilombola communities existed in the state of Goiás in 2019 [12]. In 2017, 309 settlements were registered in Goiás [13].

### 2.3. Participants

The study’s target population was children born from January 2015 to December 2017, living in settled communities or traditional communities of quilombola descendants in the state of Goiás. Children reported by the head of the household as not living in the home were excluded from the study.

### 2.4. Sampling

Sampling for the SanRural Project was carried out in multiple stages. Initially, municipalities with one or more certified and recognized quilombola community in the state of Goiás were included, and information was checked in the official sources of accreditation [14]. Therefore, of the municipalities in Goiás (*n* = 246), 45 (18.3%) met this criterion and were included in the study. In addition, in these 45 municipalities, all communities of recognized settlements were included [15]. Thus, this study included all quilombola communities and settlements in the selected municipalities, representing 44 quilombola communities and 62 settlements, totaling 106 communities. Municipalities and communities were selected based on community certification criteria.

Next, the SanRural Project encompassed the following sampling units: (i) families and (ii) individuals. Families were selected by systematic random sampling. In each community, the first individual was selected by simple random sampling, and for every two households (k = 2), one family was interviewed until reaching the sample size. The sample calculation parameters for the SanRural Project study were considered, so the estimates of proportions of the leading indicators were obtained with 95% Confidence Intervals, a maximum margin of error per community of 10%, and a margin of error for the totality of communities of the same type of 2%. After selecting the family, vaccination card information was collected from all individuals in the household, including the children. Thus, all eligible children from the selected family were included in the study. Since the family was selected by systematic random sampling, we considered this sampling unit as the primary sampling unit (PSU) and the individuals as the secondary sampling unit (SSU).

In this study, we used data only from children born from January 2015 to December 2017. Information from children in 36 municipalities (80% of the SanRural Project municipalities), 44 settled communities (71% of the total SanRural Project settlements), and 37 quilombola communities (84.1% of the total quilombola communities in the SanRural Project) were included. Thus, data from 81 communities were analyzed, including information from 227 children (94 from settlements and 133 from quilombola communities).

**Figure 1:**
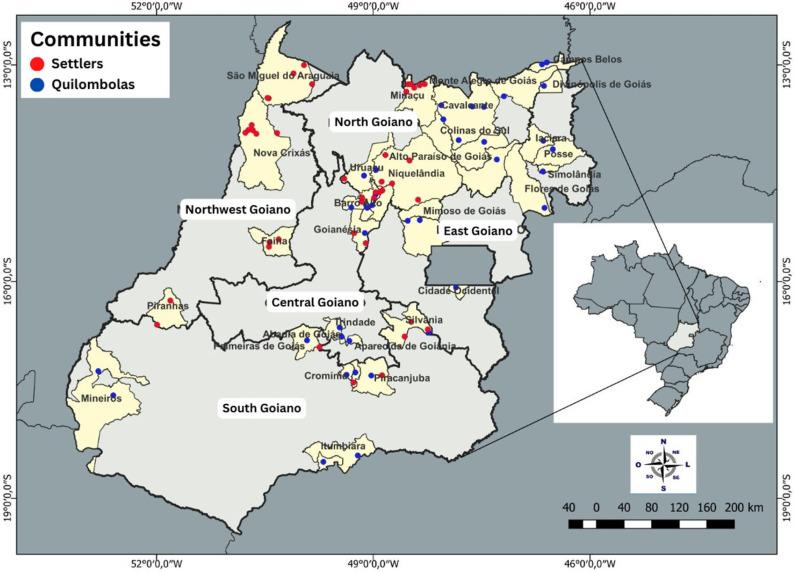
Distribution of communities and municipalities according to the mesoregions of the state of Goiás. Note: Map made using ArcGIS, version 3.24.3.

Figure 1 shows the distribution map of communities and municipalities according to the mesoregions of the state of Goiás.

A field team collected data from February 2018 to September 2019, conducting interviews on portable computers. The person responsible for the family, aged over 18, was asked to answer the research questions in each residence.

The electronic instrument for data collection contained questions about the family’s socioeconomic status, housing conditions, and the health characteristics of household residents. In addition, at the time of the interviews, the vaccination cards of all household residents were photographed.

The study included a total of 227 children distributed across settler communities (*n* = 94) and quilombola communities (*n* = 133). For children who did not present their vaccination cards during the interview (*n* = 80), vaccination data were obtained from the Information System of the National Immunization Program (SI-PNI) in Brazil. Of the investigated children (*n* = 227), 23 had no vaccination records and were considered unvaccinated [16].

### 2.5. Variables

The construction of variables related to vaccination was based on definitions supported by Brazil’s National Immunization Program recommendations and the World Health Organization [4,17].

Vaccines recommended and distributed free of charge by the Brazilian government for children under one year: BCG (single dose), Hepatitis B (4 doses), Rotavirus (2 doses), pentavalent DTP/Hib/Hepatitis B (3 doses), Polio (3 doses), PCV10 (2 doses), MenC (2 doses), and YF (single dose), offering protection against more than 11 diseases [17].

Doses: We considered the recommended doses according to the national child vaccination schedule for the first year, without considering the interval between doses. For multiple-dose vaccines, the record of the last dose was considered [17].

Complete basic vaccination schedule: Defined as the doses of vaccines recommended for the first year established by the basic vaccination schedule in force and applied up to 11 months and 29 days, including one dose of BCG vaccine (Bacillus Calmette-Guérin), the last dose of Hepatitis B vaccine, the last dose of Diphtheria-Tetanus-Pertussis vaccine (DTP), the last dose of Hemophilus influenzae type b vaccine (Hib), the last dose of Poliovirus vaccine (Polio), the last dose of 10-valent pneumococcal vaccine, the last dose of Rotavirus vaccine, the last dose of Serogroup C meningococcal conjugate vaccine, and one dose of Yellow Fever vaccine [17].

Incomplete basic vaccination schedule: Not receiving at least one of the doses described in the complete basic vaccination schedule.

Table 1 presents the changes made to the National Vaccination Calendars of the National Immunization Program in Brazil of the vaccines recommended for the first year between 2015 and 2017.

**Table 1:** Changes made to the National Vaccination Calendars of the National Immunization Program in Brazil of vaccines recommended for the first year between 2015 and 2017.

Vaccines	Schedule	Years		
		2015	Second Semester/2016	2017
BCG	1 dose	-	-	-
DTP	2, 4, and 6 months			
Hib	2, 4, and 6 months			
Rotavirus	2 and 4 months	-	-	-
YF	9 months	-	-	-
Hepatitis B	At birth, 2, 4, and 6 months	-	-	-
MenC	3 and 5 months, booster 15 months	-	3 and 5 months, booster 12 months	-
Polio	2 and 4 months (IPV), 6 months (OPV)	-	2, 4, and 6 months (IPV)	-
PCV10	2, 4, and 6 months, booster 12 months	-	2 and 4 months, booster 12 months	-

BCG: Bacillus Calmette-Guérin vaccine; DTP: Diphtheria-Tetanus-Pertussis vaccine; Hib: *Haemophilus influenzae* type b vaccine; Polio: Poliovirus vaccine (inactivated Polio vaccine (IPV))/oral Polio vaccine (OPV)); PCV10: 10-valent pneumococcal vaccine; MenC: Serogroup C meningococcal conjugate vaccine; YF: Yellow Fever vaccine. Source: Ordinances and technical reports of the National Immunization Program [18,19,20,21,22]. Note: In this study, the recommended vaccine doses up to 11 months and 29 days were included; therefore, the first dose of the measles-mumps-rubella (MMR) vaccine and the booster of the PCV10 vaccine were not considered.

General vaccine coverage: Vaccination coverage (VC) was calculated according to the applied doses and was defined as the percentage of children with a complete basic vaccination schedule, namely:

$$\mathrm{VC}\;=\frac{\mathrm{number}\;\mathrm{of}\;\mathrm{children}\;\mathrm{with}\;\mathrm{vaccination}\;\mathrm{records}\;\mathrm{and}\;\mathrm{complete}\;\mathrm{basic}\;\mathrm{vaccination}\;\mathrm{schedule}}{\mathrm{total}\;\mathrm{number}\;\mathrm{of}\;\mathrm{children}\;\mathrm{with}\;\mathrm{vaccination}\;\mathrm{records}}\times 100$$

The dependent variable in this study was incomplete general vaccination coverage related to the vaccination situation (yes or no) according to the applied doses described in the basic vaccination schedule, evaluated at 11 months and 29 days.

The independent variables included the sex of the child (male or female), the type of community (settlement or quilombola), housing zone (rural or urban/periurban), mesoregion of Goiás (Central Goiano, East Goiano, Northwest Goiano, North Goiano, or South Goiano), mother’s age (≤28 or ≥29 years), number of people in the house (≤5 or ≥6), internet access (yes or no), income (≤USD 277.91 or ≥ USD 277.92), health professional visit in the last year (yes or no), and community healthcare unit availability (yes or no). Quantitative variables, such as the mother’s age, number of people in the house, and income, were categorized based on their mean (less than or equal to the mean versus greater than or equal to the mean).

### 2.6. Statistical Analysis

The data collected during the interview, information about the vaccines recorded on the vaccination card, and the vaccine data obtained from the *SI-PNI* were exported to statistical analysis software (IBM SPSS^®^, version 24 and StataCorp. 2021. Stata Statistical Software: Release 17. College Station, TX, USA: StataCorp LLC.).

All analyses were performed using the complex sample design. Stata’s “survey” package was used. The selected families were included as PSU, and the type of community (settlement/quilombola communities) was used as a stratum. Individual selection sample weights were included for each child [23], considering the selection probability according to their community, sex, and age group.

A descriptive analysis of the participants’ characteristics was carried out initially, followed by Pearson’s chi-square test corrected for design to assess differences in characteristics between children from settlements and communities. Estimates of the coverage of the complete immunization schedule by type of vaccine and type of community were then calculated, along with 95% Confidence Intervals (95%CI). Next, bivariate and multiple analyses were performed using binary logistic regression to identify the factors associated with incomplete general vaccination coverage. In the bivariate analysis, the dependent variable was associated with each of the independent variables analyzed. Next, the variables that presented a *p*-value < 0.25 were included in the multiple logistic regression model, single input method. The magnitude of the multiple analysis effect was presented as Adjusted Odds Ratios (AOR) and 95%CI. Variables with *p*-values < 0.05 were considered significantly associated with the outcome.

### 2.7. Ethical Aspects

The SanRural Project was approved by the Research Ethics Committee of the Federal University of Goiás (CAAE number 2.886.174/2018). All participants signed the Terms of Free and Informed Consent applied to the family by signature or fingerprint of the interviewee.

## 3. Results

In the investigated communities, there were 227 children born between 2015 and 2017, with 94 (41.4%) from settlements and 133 (58.6%) from quilombola communities.

Population Characteristics:

Table 2 shows the characteristics of the participants by type of community. Of the total children included in the study (*n* = 227), 56.7% were male and 43.3% were female. Regarding the children’s mothers, 63.2% were aged 28 or younger. Concerning the children’s families, 66.4% had five people or fewer, 53.0% had access to the internet, and 61.5% had a gross income of less than or equal to USD 277.91. Furthermore, most children lived in quilombola communities (*n* = 133; 58.6%), rural areas (*n* = 172; 75.8%), and municipalities located in the North Goiano region (*n* = 77; 36.8%). As for the characteristics of access to health services for the children’s families, it was identified that, in the last year, 59.9% received a visit from a health professional and 66.2% of the communities where the children lived did not have a public health unit.

After a global evaluation of the variables, a statistical difference was observed between the communities (*p* < 0.05) concerning the following characteristics: area of residence, mesoregion, number of people in the home, access to the internet, and the existence of a public health unit in the community (*p* = 0.000).

### 3.1. Vaccination Coverage

Table 3 presents the vaccination coverage of the basic vaccination schedule for the first year evaluated at 11 months and 29 days. The overall vaccination coverage at 11 months and 29 days was 52.8% (95% CI: 45.5–59.9%). By community, the general vaccination coverage for the first year was 63.6% (95% CI: 51.7–74.1%) for settler communities and 48.0% (95% CI: 39.3–56.9%) for quilombola communities. The vaccine coverage by the investigated vaccine ranged from 70.4% for the Yellow Fever vaccine to 78.3% for the Rotavirus vaccine.

### 3.2. Factors Associated with Incomplete General Immunization Coverage

The binary logistic regression model was adjusted for the child’s sex, type of community, housing zone, mesoregion, number of people in the house, and health professional visits in the last year. These variables had a *p*-value of less than 0.25 in the bivariate analysis. Based on the multiple analysis, it was observed that the odds of an incomplete vaccination schedule were higher in children who had not received a visit from a health professional in the last year (AOR: 1.96; 95%CI: 1.03–3.73) compared to those who had received such visits (Table 4).

## 4. Discussion

In Brazil, information on the health and vaccination status of racial/ethnic minorities and rural groups is still scarce [10,24,25]. Therefore, this study presents the first information regarding vaccination coverage for children in rural settlements and quilombola communities in Goiás.

The present study showed a predominance of children from low-income families. However, investigations on these populations also suggest a predominance of disadvantaged groups with characteristics that make them individually, socially, and programmatically vulnerable [24,26,27].

This study identified low overall vaccination coverage, a relevant indicator of this population’s precarious living and health conditions. While the World Health Organization encourages all countries to achieve global immunization coverage greater than or equal to 90% for vaccines regulated by the country [28], the present study showed an overall vaccination coverage of 52.8% (95%CI: 45.5–59.9%). It is essential to highlight that no statistical differences were observed between general vaccination coverage stratified by the investigated community (settlers and quilombolas).

In Brazil, investigations in urban municipalities also showed higher vaccination coverage in children compared to the present study’s general vaccination coverage [29]. Indeed, the last immunization coverage survey in urban areas was carried out in the country in 2007 and evaluated immunization coverage for vaccines recommended in the first year, including a dose of the MMR vaccine. A total of 17,149 children from 26 Brazilian state capitals and the Federal District were investigated and had complete vaccination coverage of 81.0% (95%CI: 80.4–81.6%) at 18 months [29], which is about 1.5 times greater than the general vaccination coverage of the present study.

Garcia et al. [30] conducted a study in a medium-sized municipality in the Southeast Region of Brazil and analyzed the vaccination coverage of the complete schedule at 12 months in children born in 2015. The result was a coverage of 77.1% (95%CI: 72.6–81.0%). Similar data were also identified in a study in the southern region of Brazil, which showed vaccination coverage for the complete basic vaccination schedule (one dose of BCG, one dose of SCR, three doses of Polio, and three doses of pentavalent) among children born in 2015 to be 77.2% (95% CI: 75.8–78.4%) [31].

At the international level, wide variations in general immunization coverage have been observed in different regions worldwide. In African countries, immunization coverage for recommended vaccines during the first year was estimated at 29.7% in Ethiopia and 67.6% in Senegal [32,33]. In India, among children aged 12 to 36 months residing in rural areas of 26 states, complete immunization coverage, i.e., one dose of the BCG vaccine, three doses of the DTP vaccine, and one dose of the measles vaccine, was 53.2% (95% CI: 52.7–53.7%) [34].

In developed countries such as the United States and China, recent investigations have revealed specific differences in vaccine coverage. For example, a national survey conducted in the United States in 2017 found that vaccination coverage for children aged 19 to 35 months living in rural areas was 66.8% (95% CI: 63.6–69.9%) for the complete schedule of vaccines (acellular DTP, Polio, SCR, Hib, Hepatitis B, varicella, and pneumococcal) [35]. In China, data from 2016 showed that 94.0% (95%CI: 91.4–95.9%) of children aged 24 to 35 months living in rural areas were fully vaccinated with scheduled vaccines for the first year (BCG, Hepatitis B, Polio, DTPa, and measles and rubella (MR)) [36].

These inequalities in vaccination coverage can be explained by the diversity of vaccines recommended in each country’s vaccination schedules, making vaccination programs and schemes more complex [5]. In addition, of course, these economic, social, and health discrepancies exist worldwide. It is important to remember that, as of 2016, underdeveloped countries such as Senegal, Ethiopia, and India began to receive financial resources from Gavi, The Vaccine Alliance, to introduce and increase vaccine access for thousands of children [37].

When evaluating vaccination coverage for each vaccine, none reached the recommended minimum coverage of 90%. While the Yellow Fever vaccine had the lowest coverage of 70.4%, the Rotavirus vaccine had the highest coverage of 78.3%. This result may be related to the immunization program’s recommended age for these vaccines. In Brazil, the Rotavirus vaccine is recommended earlier, at 2 and 4 months, while the Yellow Fever vaccine is recommended at 9 months [17]. Studies have shown greater adherence to vaccination in the first months, as vaccination dates correspond to the child’s routine consultation, which happens monthly in the first six months [38,39].

In the present study, vaccination coverage was associated with the health services offered to the investigated population. Families that did not receive a home visit from a health professional in the last year had odds of having incompletely vaccinated children that were 1.96 times higher than those who received a visit from a healthcare worker.

Brazil’s national primary care policy is crucial in discussing these data since the results are linked to the Family Health Strategy, which significantly reorganized Primary Health Care in the Unified Health System. In Brazil, one of the primary objectives of the Family Health Strategy Program (FHS-ESF) is to provide comprehensive, accessible, and continuous care with resolvability and good quality at public health units and homes through a multidisciplinary team [40,41]. In the present study, home visits seem to contribute to increased vaccination coverage of the investigated children. Furthermore, this interactive healthcare technology identifies susceptible groups in a differentiated and equitable way, promoting health education actions [42].

Although public policies in Brazil have positively impacted vaccination coverage in this study, the results show a low vaccination coverage panorama for children from racial/ethnic minorities and rural groups. Therefore, health services must be rethought for difficult-to-access groups with unique cultural characteristics. We believe it is necessary to understand the reasons for vaccine hesitancy in these groups and that creating bonds and security should be the first step towards effective health actions.

Finally, it is necessary to consider some limitations of this investigation. The SanRural Project is a household survey to investigate the health and sanitation situation of the rural and traditional populations in the state of Goiás. Therefore, other determinants to assess the factors associated with vaccine incompleteness were not investigated. Although participant compliance was high, the response rate was not measured. More studies are encouraged to address this knowledge gap in these vulnerable groups. Another limitation was the absence of some vaccination cards during data collection. However, to increase the veracity of the analysis of information on vaccination coverage, all means of searching for vaccine data were accessed from public agencies in Brazil. Another relevant point was the long period of data collection, but it is important to highlight the great difficulty that exists in accessing these groups, as they live in rural regions with difficult geographic mobility. Only quilombola communities recognized by responsible bodies in Brazil participated in this study, which restricted the participation of other communities that are in the certification process. However, we believe that the characteristics of the communities not included are similar to those that were studied, as both are located in the same geographic region, share the same public health policies, and have the same challenges inherent to the traditional population of Brazil.

## Figures and Tables

**Table 2:** Sociodemographic characteristics according to the type of community of 227 settled and quilombola children in the state of Goiás, 2015–2017.

Variables	Total	Settlers	Quilombolas	$\chi^{2}\;*$	*p-*Value
	*n* = 227	*n* = 94	*n* = 133		
Sex, *n* (%)					
Male	126 (56.7)	52 (54.0)	74 (57.9)	0.254	0.615
Female	101 (43.3)	42 (46.0)	59 (42.1)		
Housing zone, *n*(%)					
Urban/Periurban	55 (24.2)	0 (0.0)	55 (44.8)	44.272	<0.001
Rural	172 (75.8)	94 (100.0)	78 (55.2)		
Mesoregion, *n* (%)					
Central Goiano	47 (21.0)	19 (18.8)	28 (21.9)	20.719	<0.001
East Goiano	32 (14.0)	0 (0.0)	32 (20.0)		
Northwest Goiano	36 (12.9)	36 (42.6)	0 (0.0)		
North Goiano	77 (36.8)	27 (28.5)	50 (40.5)		
South Goiano	35 (15.3)	12 (10.1)	23 (17.6)		
Mother’s age (years), *n* (%)					
≤28 years	141 (63.2)	62 (65.6)	79 (62.2)	0.202	0.654
≥29 years	86 (36.8)	32 (34.4)	54 (37.8)		
Number of people in the house, *n* (%)					
≤5 people	158 (66.4)	77 (79.6)	81 (60.7)	5.742	0.017
≥6 people	69 (33.6)	17 (20.4)	52 (39.3)		
Has internet, *n* (%)					
Yes	120 (53.0)	41 (42.5)	79 (57.6)	3.659	0.057
No	107 (47.0)	53 (57.5)	54 (42.4)		
Income (USD) **, *n* (%)					
≤277.91	138 (61.5)	54 (56.5)	84 (63.7)	0.837	0.361
≥277.92	89 (38.5)	40 (43.5)	49 (36.3)		
Health professional visits in the last year, *n* (%)					
Yes	137 (59.9)	59 (58.0)	78 (61.0)	0.113	0.737
No	90 (40.1)	35 (42.0)	55 (39.3)		
Community public health unit, *n* (%)					
Yes	63 (33.8)	6 (6.8)	57 (45.5)	34.719	<0.001
No	164 (66.2)	88 (93.2)	76 (54.5)		

Notes: Mother’s age (years)—mean 27.9, standard deviation 6.5; number of people in the house—mean 4.9, standard deviation 1.6; income (USD)—mean 277.91, standard deviation 226.2. * Pearson’s chi-square test corrected for study design. ** Per month.

**Table 3:** Complete vaccination coverage and vaccine coverage, according to doses in the first year, evaluated at 12 months in settler and quilombola children in the state of Goiás, 2015–2017.

Vaccines	Complete Vaccine Schedule								
	General (*n* = 227)			Settler (*n* = 94)			Quilombola (*n* = 133)		
	n	%	95%CI	n	%	95%CI	n	%	95%CI
BCG	176	75.9	69.1–81.5	78	82.0	71.1–89.4	98	73.2	64.5–80.4
Hepatitis B	167	72.4	65.1–78.7	69	75.1	63.7–81.3	98	71.2	61.9–79.1
DTP	171	74.3	67.1–80.4	70	75.6	64.2–84.3	101	73.8	64.5–81.3
Hib	167	72.4	65.1–78.7	69	75.1	63.7–83.9	98	71.2	61.9–79.1
Polio	171	75.8	68.9–81.6	70	75.8	64.6–84.6	101	75.7	66.8–82.9
PCV10	184	77.9	71.0–83.5	75	81.4	71.1–88.6	109	76.3	67.3–83.5
Rotavirus	177	78.3	71.6–83.7	72	79.0	68.5–86.7	105	78.0	69.3–84.7
MenC	179	78.0	71.0–83.5	75	81.4	71.1–88.6	104	76.3	67.3–83.5
YF	161	70.4	63.1–76.8	65	72.9	61.8–81.7	96	69.3	60.0–77.4
General vaccine coverage	121	52.8	45.5–59.9	54	63.6	51.7–74.1	67	48.0	39.3–56.9

BCG: Bacillus Calmette-Guérin vaccine; DTP: Diphtheria-Tetanus-Pertussis vaccine; Hib: *Haemophilus influenzae* type b vaccine; Polio: Poliovirus vaccine; PCV10: 10-valent pneumococcal vaccine; MenC: Serogroup C meningococcal conjugate vaccine; YF: Yellow Fever vaccine.

**Table 4:** Factors associated with incomplete general vaccination coverage for the first year. Goiás, Brazil, 2015–2017.

Variables	Bivariate Analysis					Multiple Analysis *	
	Vaccine Schedule (*n* = 227)						
	Total	Incomplete	Complete	*p-*Value	OR (95%CI)	*p-*Value	AOR (95%CI)
	(*n* = 227)	(*n* = 106)	(*n* = 121)				
Sex							
Male	126	66 (52.3%)	60 (47.7%)		1.00		1.00
Female	101	40 (40.6%)	61 (59.4%)	0.119	0.62 (0.34–1.13)	0.273	0.78 (0.37–1.4)
Community type							
Settler	94	40 (36.4%)	54 (63.6%)		1.00		1.00
Quilombola	133	66 (52.0%)	67 (48.0%)	0.040	1.89 (1.03–3.48)	0.882	1.08 (0.40–2.89)
Housing zone							
Rural	172	75 (40.3%)	97 (59.7%)		1.00		1.00
Urban/periurban	55	31 (62.6%)	24 (37.4%)	0.008	2.48 (1.28–4.80)	0.092	2.28 (0.87–5.92)
Mesoregion							
Central Goiano	47	28 (61.2%)	19 (38.8%)		1.00		1.00
East Goiano	32	10 (33.9%)	22 (66.1%)	0.027	0.32 (0.12–0.88)	0.122	0.43 (0.15–1.26)
Northwest Goiano	36	13 (30.1%)	23 (69.9%)	0.014	0.27 (0.09–0.76)	0.132	0.40 (0.12–1.32)
North Goiano	77	38 (49.1%)	39 (50.9%)	0.241	0.61 (0.27–1.39)	0.726	0.84 (0.32–2.24)
South Goiano	35	17 (50.4%)	18 (49.6%)	0.371	0.64 (0.25–1.69)	0.370	0.63 (0.23–1.74)
Mother’s age (years)							
≤28	141	73 (51.2%)	68 (48.8%)		1.00		1.00
≥29	86	33 (40.4%)	53 (59.6%)	0.156	0.65 (0.35–1.18)	0.101	0.58 (0.30–1.11)
Number of people in the house							
≤5	158	68 (43.4%)	90 (56.6%)		1.00		1.00
≥6	69	38 (54.8%)	31 (45.2%)	0.148	1.58 (0.84–2.93)	0.092	1.82 (0.90–3.65)
Has internet							
Yes	120	56 (46.8%)	64 (53.2%)		1.00		
No	107	50 (47.8%)	57 (52.2%)	0.889	1.04 (0.58–1.87)		
Income (USD)							
≤277.91	138	68 (47.8%)	70 (52.2%)		1.00		
≥277.92	89	38 (46.3%)	51 (53.7%)	0.847	0.94 (0.51–1.73)		
Health professional visit in the last year							
Yes	137	52 (40.8%)	84 (59.2%)		1.00		1.00
No	90	54 (56.9%)	37 (43.1%)	0.035	1.91 (1.05–3.49)	0.039	1.96 (1.03–3.73)
Is a community healthcare unit available?							
Yes	63	29 (50.8%)	34 (49.2%)		1.00		
No	164	77 (45.5%)	87 (54.5%)	0.505	0.80 (0.43–1.52)		

Note: Incomplete and complete vaccination coverage is presented as *n* (%), where *n* is the number of observations in the sample and % is the percentage weighted by the complex sampling design. AOR: Adjusted Odds Ratio; 95.0%CI: 95% Confidence Interval; OR: Odds Ratio. * Binary logistic regression model adjusted for child’s gender, type of community, housing zone, mesoregion, number of people in the house, and health professional visit in the last year.

## Data Availability

The datasets used and/or analyzed during the current study are available from the corresponding author upon reasonable request.
